# APOBEC Mutational Signature and Tumor Mutational Burden as Predictors of Clinical Outcomes and Treatment Response in Patients With Advanced Urothelial Cancer

**DOI:** 10.3389/fonc.2022.816706

**Published:** 2022-03-07

**Authors:** Divya Natesan, Li Zhang, Henry J. Martell, Tanya Jindal, Patrick Devine, Bradley Stohr, Carlos Espinosa-Mendez, James Grenert, Jessica Van Ziffle, Nancy Joseph, Sarah Umetsu, Courtney Onodera, Michelle Turski, Emily Chan, Arpita Desai, Rahul Aggarwal, Anthony Wong, Sima Porten, Jonathan Chou, Terence Friedlander, Lawrence Fong, Eric J. Small, Alejandro Sweet-Cordero, Vadim S. Koshkin

**Affiliations:** ^1^ Helen Diller Family Cancer Center, University of California San Francisco, San Francisco, CA, United States; ^2^ Department of Pediatrics, Benioff Children’s Hospital, University of California, San Francisco, San Francisco, CA, United States

**Keywords:** bladder cancer, APOBEC mutational signature, tumor mutational burden, next-generation sequencing, urothelial cancer, hypermutated, biomarkers, immune checkpoint inhibitor (ICI)

## Abstract

**Introduction:**

Tumor mutational burden (TMB) and APOBEC mutational signatures are potential prognostic markers in patients with advanced urothelial carcinoma (aUC). Their utility in predicting outcomes to specific therapies in aUC warrants additional study.

**Methods:**

We retrospectively reviewed consecutive UC cases assessed with UCSF500, an institutional assay that uses hybrid capture enrichment of target DNA to interrogate 479 common cancer genes. Hypermutated tumors (HM), defined as having TMB ≥10 mutations/Mb, were also assessed for APOBEC mutational signatures, while non-HM (NHM) tumors were not assessed due to low TMB. The logrank test was used to determine if there were differences in overall survival (OS) and progression-free survival (PFS) among patient groups of interest.

**Results:**

Among 75 aUC patients who had UCSF500 testing, 46 patients were evaluable for TMB, of which 19 patients (41%) had HM tumors and the rest had NHM tumors (27 patients). An additional 29 patients had unknown TMB status. Among 19 HM patients, all 16 patients who were evaluable for analysis had APOBEC signatures. HM patients (N=19) were compared with NHM patients (N=27) and had improved OS from diagnosis (125.3 months *vs* 35.7 months, p=0.06) but inferior OS for patients treated with chemotherapy (7.0 months *vs* 13.1 months, p=0.04). Patients with APOBEC (N=16) were compared with remaining 56 patients, comprised of 27 NHM patients and 29 patients with unknown TMB, showing APOBEC patients to have improved OS from diagnosis (125.3 months *vs* 44.5 months, p=0.05) but inferior OS for patients treated with chemotherapy (7.0 months *vs* 13.1 months, p=0.05). Neither APOBEC nor HM status were associated with response to immunotherapy.

**Conclusions:**

In a large, single-institution aUC cohort assessed with UCSF500, an institutional NGS panel, HM tumors were common and all such tumors that were evaluated for mutational signature analysis had APOBEC signatures. APOBEC signatures and high TMB were prognostic of improved OS from diagnosis and both analyses also predicted inferior outcomes with chemotherapy treatment.

## Introduction

Urothelial carcinoma (UC) is a common malignancy with treatment options that have advanced significantly in recent years. Most patients are initially diagnosed with non-muscle-invasive bladder cancer (NMIBC) or muscle-invasive bladder cancer (MIBC) but many unfortunately progress to metastatic disease. Patients diagnosed with MIBC in the absence of distant metastases are treated with cisplatin-based neoadjuvant chemotherapy (1). Patients with metastatic or advanced urothelial cancer (aUC), who have distant metastases outside the organ where tumor originated, are generally considered to have incurable disease. However, many options to delay progression and extend survival are available including chemotherapy, immunotherapy (IO), and now increasingly targeted agents as well. The typical 1^st^ line standard of care (SOC) treatment is platinum-based chemotherapy, followed by IO as 2^nd^ line at the time of progression or as switch maintenance therapy. Enfortumab vedotin (EV) is now approved following progression on at least one prior line of therapy and has phase III data supporting its use following progression on platinum-based chemotherapy and IO (2). Sacituzumab govitecan also recently received accelerated approval in treatment-refractory aUC (3).

In recent years this has been a dynamic treatment landscape with a variety of agents and combination therapies being investigated in clinical trials. This rapid expansion of treatment options highlights the need for novel prognostic and predictive biomarkers in aUC since decisions need to be made among multiple available treatment options. These are frequently very consequential decisions as the rate of patient attrition with each successive therapy is high (4). Next-generation sequencing (NGS) and other biomarkers such as tumor mutational burden (TMB), tumor PD-L1 expression and microsatellite instability (MSI) are frequently utilized to make decisions about treatments (5, 6). NGS to identify specific mutations in aUC patients is especially important as one agent in particular, erdafitinib, is now approved for a biomarker selected population of patients with *FGFR3* alterations (7).

Numerous NGS platforms are commercially available, but many institutions also have proprietary institutional panels that can detect clinically-relevant alterations. At the University of California, San Francisco (UCSF) the UCSF500 panel utilizes tumor tissue samples to detect 479 oncologic genes and select introns of 47 genes. NGS panels, including UCSF500, can also define TMB which can potentially serve as an important biomarker. Previous analyses have shown that increased TMB is prognostic of longer overall survival (OS) in UC regardless of treatment given (8). High TMB was also associated with improved responses in patients treated with IO in aUC clinical trials, including nivolumab and atezolizumab (9–11). Pembrolizumab has additionally received a tumor-agnostic approval for patients whose tumors have high TMB based on observed clinical benefit (12). On the other hand, the role of TMB as a predictive marker for chemotherapy response is still being explored and available data has not been conclusive (13). Additional prognostic and predictive information may be available from assessment of other biomarkers, including the presence of apolipoprotein B mRNA editing catalytic polypeptide-like (APOBEC) enzyme mutational signature. APOBEC family of enzymes function as cytosine deaminases with a likely role in antiretroviral defense. In bladder tumors and other malignancies they are likely responsible for hypermutation and contribute to cancer mutagenesis (14, 15). Tumors harboring APOBEC mutational signature have been shown to have a higher TMB (16). Previously reported data also suggests that presence of APOBEC signature is prognostic of improved outcomes (17), and may be associated with improved responses to immunotherapy (9, 16). The association of APOBEC mutational signature with chemotherapy responses has not been explored as extensively.

Leveraging data from UCSF500 tumor testing in patients with advanced bladder cancer, we performed a retrospective analysis assessing the prognostic and predictive value of TMB and APOBEC mutational signature in this patient population. We hypothesized that presence of both TMB and APOBEC would be associated with more favorable clinical outcomes and would be predictive of improved responses to immunotherapy while not having predictive value in assessing potential response to chemotherapy.

## Materials and Methods

### Study Design

This was a single-institution, retrospective study assessing clinical and treatment outcomes in urothelial carcinoma patients whose tumor tissue was assessed with UCSF500, an institutional NGS assay. Eligible patients had to meet the following criteria: 1) have a pathologically documented diagnosis of bladder cancer, upper tract urothelial cancer or urethral cancer, 2) have available UCSF500 results from a tumor biopsy sample, and 3) have demographic, clinical, and treatment data available for abstraction from the electronic medical record (EMR). The data for this retrospective review were collected for eligible patients under a UCSF Institutional Review Board (IRB) approved protocol. A total of 75 patients with clinical and treatment data spanning the time period from April 2016 through April 2020 were included in the analysis.

### Molecular Analyses

NGS results from tumor samples included in this analysis were obtained using the institutional UCSF500 panel developed and utilized at UCSF. The UCSF500 Cancer Gene Test uses capture-based next-generation sequencing to target and analyze the coding regions of 479 cancer genes, as well as select introns of 47 genes. As part of this test, genomic DNA is extracted from paraffin embedded tumor tissue and a paired normal tissue sample (paraffin embedded or blood sample) if available. Target enrichment is performed by hybrid capture using custom oligonucleotides. Sequencing of captured libraries is performed on an Illumina HiSeq 2500 by a CLIA-certified laboratory: UCSF Genomic Sequencing Services Lab at Institute for Human Genetics (San Francisco, CA). Sequence reads are de-duplicated to allow for accurate allele frequency determination and copy number calling. The analysis uses open source or licensed software for alignment to the human reference sequence UCSC build hg19 (NCBI build 37) and variant calling. Additionally, microsatellite instability analysis is performed with MSIsensor (18).

Patients who had paired tumor and normal tissue samples available were also potentially assessed for TMB and APOBEC mutational signature analysis. Patients who only had tumor samples (no normal samples) were not considered evaluable for TMB or APOBEC as there was no normal sample to remove germline variants. Among patient samples assessed for TMB, hypermutated samples were defined as those with ≥10 mutations per megabase of DNA sequenced (19). Mutational signatures were extracted from the panel sequencing data using deconstructSigs (20). Only samples with ≥50 total somatic mutations were analyzed for mutational signatures, the recommended threshold for deconstructSigs. Trinucleotide counts were normalized for the trinucleotide composition of the UCSF500 panel footprint using the “tri.counts.method” option in deconstructSigs. Version two of the COSMIC mutational signature set was used as reference (21), in addition to a cisplatin mutational signature (22), resulting in a reference set of 31 mutational signatures.

### Clinical and Response Assessment

Retrospective chart review of the EMR was undertaken for all patients included in the analysis. Collected data included patient demographics and tumor characteristics. Relevant clinical and treatment characteristics were also collected including dates of initial diagnosis and onset of metastatic disease, administered treatments and treatment responses; and final follow-up and vital status. For patients who were treated in the metastatic setting with chemotherapy or with immune checkpoint inhibitors, the dates of treatment start and finish and responses to treatment were recorded.

Response to and progression on treatment was assessed retrospectively by the chart abstractor based on available information in notes and radiographic studies. Imaging studies used to define treatment response or progression were done at the discretion of the treating provider. Radiology and pathology results were assessed based on information available in the electronic medical record; no central review was performed.

Overall survival (OS) was defined as the time from initial diagnosis until the date of death, if available. For treatment specific outcomes, observed response rate (ORR) was defined by the chart abstractor as the best response to treatment. OS was defined as the time from treatment start until the date of death, whereas progression-free survival (PFS) was defined as the time from treatment start until date of progression or date of death, whichever happened first.

### Statistical Analyses

Two major analyses were undertaken as part of this study. As part of the TMB analysis, patients whose tumors were evaluable for TMB were divided into Hypermutated (HM; TMB ≥ 10 mut/Mb) *vs*. Non-hypermutated (NHM; TMB < 10 mut/Mb) groups and outcomes were compared between these groups. As part of the APOBEC analysis, patients with hypermutated tumors which were detected to have APOBEC mutational signatures were compared to Other (non-APOBEC) patients whose tumors were not known to harbor an APOBEC mutational signature due to either being NHM or having too few mutations to be evaluable for TMB. To assess prognostic outcomes in these comparison groups, we evaluated median OS from initial diagnosis regardless of treatment administered. To assess predictive value of these biomarkers for specific treatments including chemotherapy and immune checkpoint inhibitors in aUC patients, we compared ORR to the treatment for metastatic disease, as well as median PFS and median OS from treatment initiation for metastatic disease.

Continuous variables were summarized using median and range and categorical variables were described with frequency and percent of total. Continuous variables were compared between the groups by Wilcoxon rank sum test and categorical variables were compared between the groups by Chi-squared test. The logrank test was used to assess if there were differences in OS from initial diagnosis, IO initiation, and chemotherapy initiation in addition to PFS from IO initiation and chemotherapy initiation.

## Results

### Clinical Characteristics

**Figure 1:**
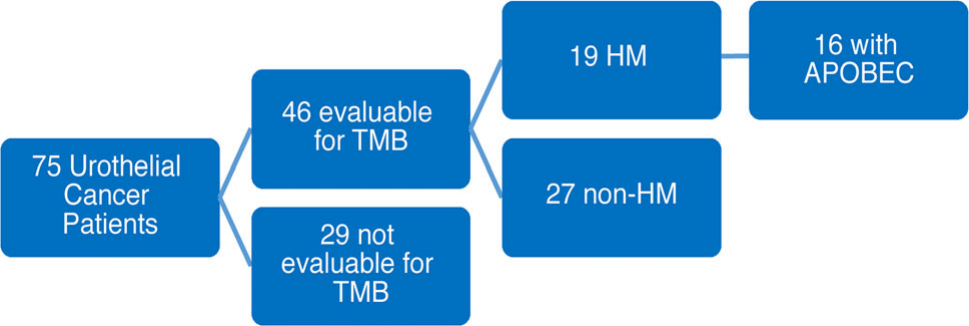
Consort Diagram for the study.

Among 75 eligible patients that underwent UCSF500 testing, 46 patients had tissue evaluable for tumor mutational burden (TMB). Of the 46 evaluable cases, 19 patients were hypermutated, while the remaining 27 patients had non-hypermutated tumors. Of the evaluable hypermutated cases, 16 were positive for APOBEC mutational signatures (**Figure 1**). The remaining 3 HM patients were found to not be evaluable for APOBEC mutational signature analysis due to insufficient number of mutations. Consequently, they were excluded from further APOBEC analysis comparisons as their APOBEC mutational status was unknown.

The demographic and clinical characteristics of patients included in the TMB and APOBEC analyses are shown in **Tables 1**, **2**, respectively. Patients included in this analysis were representative of the population with aUC, with a median age in the late 60s and the majority of patients were men. Median follow-up for all patients from initial diagnosis was 15.5 months. Most patients in this study were Caucasian but a significant minority were Asian or Hispanic/Latino, reflecting the patient population served by the UCSF Helen Diller Family Comprehensive Cancer Center. Notably, in the group of patients with APOBEC mutational signatures, there was a numerically higher percentage of patients with primary bladder tumors and of patients whose tumors had a pure urothelial histology, although this did not quite meet statistical significance. More patients with either HM or APOBEC tumors had a prior smoking history relative to the comparison groups. Most commonly observed tumor alterations were also representative of what has previously been described in the bladder tumor mutational landscape (23) and overall were fairly consistent across the two comparisons. However, fewer *FGFR3* alterations were observed in the HM or APOBEC groups than in the comparison groups; these differences were not statistically significant. Testing for PD-L1 status was limited but from the collected data, the percentages of tumors with increased PD-L1 expression were comparable across subgroups.

**Table 1:** Clinical characteristics of patients in the hypermutated and non-hypermutated groups.

TMB Analysis			
	HM (N=19)	NHM (N=27)	P-value
**Median Age at Diagnosis (Range)**	65 (50,87)	69 (40,87)	0.13
**Gender**			1.00
** Male**	12 (63%)	17 (63%)	
** Female**	7 (37%)	10 (37%)	
**Race/Ethnicity**			0.50
** Asian**	1 (5%)	1 (4%)	
** Black or African American**	0 (0%)	4 (15%)	
** Hispanic or Latino**	2 (11%)	1 (4%)	
** White**	14 (74%)	21 (78%)	
** Unknown**	2 (11%)	0 (0%)	
**Smoking History (Ever Smoker)**	13 (68%)	13 (48%)	0.29
**Primary Tumor Location**			0.29
** Bladder**	16 (84%)	20 (74%)	
** Upper Tract**	1 (5%)	5 (19%)	
** Urethra**	0 (0%)	1 (4%)	
** Multiple**	1 (5%)	0 (0%)	
**N/A**	1 (5%)	1 (4%)	
**Histology**			0.33
** Pure Urothelial**	14 (74%)	19 (70%)	
** Pure Squamous**	0 (0%)	2 (7%)	
** Squamous Component**	3 (16%)	1 (4%)	
** Small Cell / NE**	1 (5%)	2 (7%)	
** Adenocarcinoma**	0 (0%)	1 (4%)	
** Sarcomatoid**	0 (0%)	2 (7%)	
** Urachal**	1 (5%)	0 (0%)	
**Biopsy Source**			0.96
Primary Tumor*	10 (53%)	14 (52%)	
** Metastatic**	9 (47%)	13 (48%)	
**Most Common Alterations (N)**	TERT (16, 84%)	TERT (20, 74%)	N/A
	TP53 (13, 68%)	KDM6A (13, 48%)	
	KMT2D (7, 37%)	TP53 (13, 48%)	
	PIK3CA (7, 37%)	CDKN2A (8, 30%)	
	ARID1A (6, 32%)	CDKN2B (8, 30%)	
	CDKN2A (6, 32%)	FGFR3 (7, 26%)	
	ERBB2 (6, 32%)	ARID1A (6, 22%)	
	KDM6A (6, 32%)	ELF3 (5, 19%)	
	CCND1 (3, 16%)	KMT2D (5, 19%)	
	CDKN2B (3, 16%)	RB1 (5, 19%)	
	RB1 (3, 16%)		
	FGFR3 (2, 11%)		
**PD-L1 Available**	4 (21%)	10 (37%)	0.73
Positive (CPS≥10)**	2 (50%)	4 (40%)	
Negative (CPS<10)**	2 (50%)	6 (60%)	
**MSI-High Tumor**	1 (5%)	0 (0%)	0.86
**Metastatic Disease**			
** At Diagnosis**	2 (11%)	4(15%)	0.28
** Anytime during Follow-up**	13 (68%)	19 (70%)	0.89
**Definitive Surgery**	11 (58%)	19 (70%)	0.53
**Definitive Radiation Therapy**	2 (11%)	1 (4%)	0.36

HM, hypermutated; NHM, non-hypermutated; NE, neuroendocrine; MSI, microsatellite instability.

*Includes either bladder or upper tract.

**Percentages are out of available PD-L1 data.

N/A, Not Applicable.

**Table 2:** Clinical characteristics of patients in the APOBEC and Other groups.

APOBEC Analysis			
	APOBEC (N=16)	Other (N=56)	P-Value
**Median Age at Diagnosis (Range)**	65 (51, 86)	68 (31, 87)	0.66
**Gender**			0.92
** Male**	10 (62%)	38 (68%)	
** Female**	6 (38%)	18 (32%)	
**Race/Ethnicity**			0.56
** Asian**	1 (6%)	9 (16%)	
** Black or African**	0 (0%)	1 (2%)	
**Hispanic or Latino**	1 (6%)	1 (2%)	
** White**	12 (75%)	44 (79%)	
** Unknown**	2 (13%)	1 (2%)	
**Smoking History (Ever Smoker)**	11 (69%)	25 (45%)	0.16
**Primary Tumor Location**			0.08
** Bladder**	13 (81%)	38 (68%)	
** Upper Tract**	1 (6%)	15 (27%)	
** Urethra**	0 (0%)	2 (4%)	
** Multiple**	1 (6%)	0 (0%)	
** N/A**	1 (6%)	1 (2%)	
**Histology**			0.78
** Pure Urothelial**	13 (81%)	35 (63%)	
** Pure Squamous**	0 (0%)	2 (4%)	
** Squamous Component**	2 (13%)	8 (14%)	
** Small Cell / NE**	1 (6 %)	2 (4%)	
** Adenocarcinoma**	0 (0%)	3 (5%)	
** Plasmacytoid**	0 (0%)	3 (5%)	
** Micropapillary**	0 (0%)	1 (2%)	
** Sarcomatoid**	0 (0%)	2 (4%)	
**Biopsy Source**			0.75
Primary Tumor*	7 (44%)	27 (48%)	
** Metastatic**	9 (56%)	29 (52%)	
**Most Common Alterations (N)**	TERT (14, 88%)	TERT (40, 71%)	N/A
	TP53 (10, 63%)	TP53 (31, 55%)	
	KMT2D (6, 38%)	CDKN2A (20, 36%)	
	ARID1A (6, 38%)	KDM6A (19, 34%)	
	ERBB2 (6, 38%)	CDKN2B (18, 66%)	
	KDM6A (6, 38%)	ARID1A (12, 21%)	
	CDKN2A (5, 31%)	FGFR3 (11, 20%)	
	PIK3CA (5, 31%)	RB1 (11, 20%)	
	CCND1 (3, 19%)	KMT2D (10, 18%)	
	CDKN2B (3, 19%)	ERBB2 (9, 16%)	
	RB1 (3, 19%)		
	FGFR3 (2, 13%)		
**PD-L1 Available**	4 (25%)	24 (43%)	0.63
Positive (CPS≥10)**	2 (50%)	15 (63%)	
Negative (CPS<10)**	2 (50%)	9 (37%)	
**MSI-High Tumor**	1 (6%)	0 (0%)	0.50
**Metastatic Disease**			
** At Diagnosis**	1 (6%)	11(20%)	0.28
** Anytime During Follow-up**	10 (63%)	40 (71%)	0.49
**Definitive Surgery**	10 (63%)	36 (64%)	1
**Definitive Radiation Therapy**	2 (13%)	1 (2%)	0.06

HM, hypermutated; NHM, non-hypermutated; NE, neuroendocrine; MSI, microsatellite.

*Includes either bladder or upper tract.

**Percentages are out of available PD-L1 data.

N/A, Not Applicable.

### TMB Analysis

**Figure 2:**
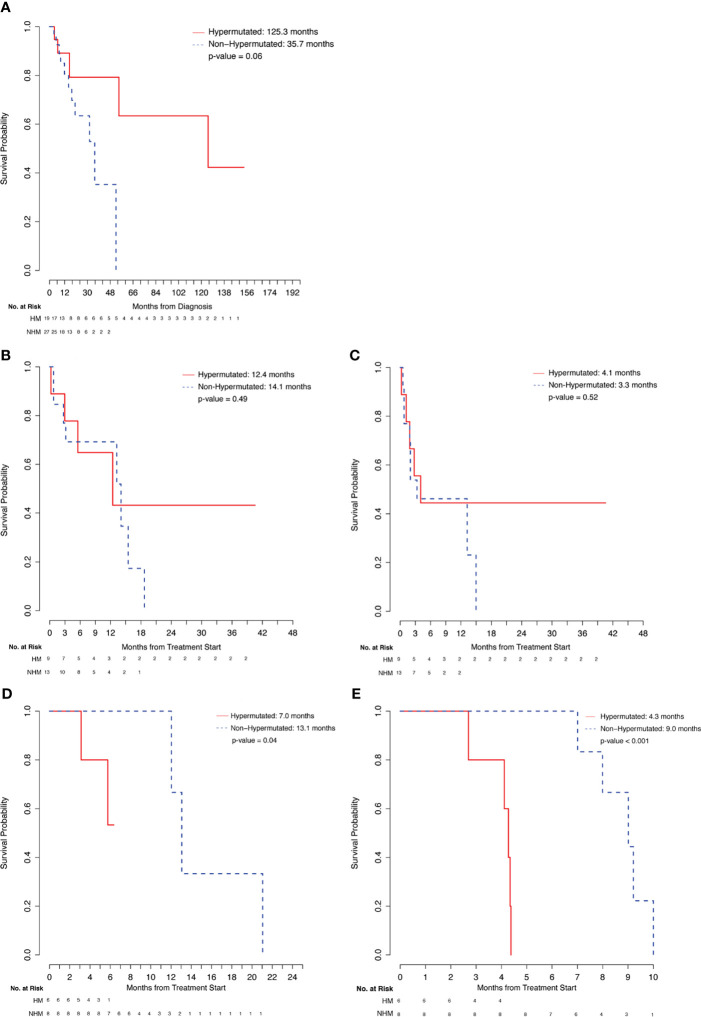
Comparison of hypermutated patients. **(A)** Overall Survival from Diagnosis. **(B)** Overall Survival from Immunotheraphy Start. **(C)** Progression-Free Survival from Immunotherapy Start. **(D)** Overall survival from Chemotherapy Start. **(E)** Progression-Free Survival from Chemotherapy Start.

In the comparison of outcomes for 19 patients with hypermutated tumors (TMB ≥ 10 mut/Mb) and 27 patients with non-hypermutated tumors (TMB < 10 mut/Mb), HM patients had longer median OS (125.3 months *vs* 35.7 months, p = 0.06) from initial diagnosis, suggesting HM status to be a positive prognostic marker (**Figure 2A** and **Table 3**). At the time of data cutoff, 74% of HM patients and 59% of NHM patients were alive. A total of 9 HM patients were treated with IO, and 6 HM patients were treated with chemotherapy.

**Table 3:** Comparison of outcomes in hypermutated vs non-hypermutated patients.

HM vs. NHM			
Patient Group (N)	Comparison	Median Survival (months)	p-value
HM (19) vs NHM (27)	OS from initial diagnosis	125.3 vs 35.7	0.06
	From IO Start		
HM (9) vs	OS	12.4 vs 14.1	0.49
NHM (13)	PFS	4.1 vs 3.3	0.52
	From Chemotherapy Start		
HM (6) vs	OS	7.0 vs 13.1	**0.04**
NHM (8)	PFS	4.3 vs 9.0	**<0.001**

HM, hypermutated; NHM, non-hypermutated; OS, overall survival; PFS, progression-free survival; IO, immunotherapy.

Bold: statistically significant.

In comparing outcomes with IO treatment, no significant differences were observed for the 9 HM patients relative to the 13 NHM patients who received treatment with an immune checkpoint inhibitor (**Figures 2B, C** and **Table 3**). HM patients were treated with Pembrolizumab (6/9, 33%) and Atezolizumab (3/9, 33%) and NHM patients were treated with Pembrolizumab (8/13, 62%), Atezolizumab (3/13, 23%), Nivolumab/Ipilimumab (1/13, 8%), and Durvalumab/Tremelimumab (1/13, 8%). Numerically higher ORR was observed in the HM group among evaluable patients (4/8, 50%) relative to the NHM group (4/12, 33%), although this difference was not statistically significant. Similarly, median PFS (4.1 *vs* 3.3 months, p=0.52) and OS (12.4 *vs* 14.1 months, p=0.49) from treatment start for the two groups were comparable.

On the other hand, in comparing outcomes with platinum-based chemotherapy treatment for the 6 HM patients and 8 NHM patients, response rates for evaluable HM patients (3/5, 60%) and NHM patients (5/8, 63%) were similar. Importantly, HM patients were found to have shorter PFS (4.3 *vs* 9.0 months, p<0.001) and OS (7.0 *vs* 13.1 months, p=0.04) relative to the NHM patients (**Figures 2D, E** and **Table 3**). Chemotherapy treatment received by HM patients included cisplatin-based chemotherapy (4/6, 67%), carboplatin-based chemotherapy (1/6, 17%), and FOLFOX (1/6, 17%) while NHM patients received carboplatin-based chemotherapy (4/8, 50%) and cisplatin-based chemotherapy (4/8, 50%).

### APOBEC Analysis

**Figure 3:**
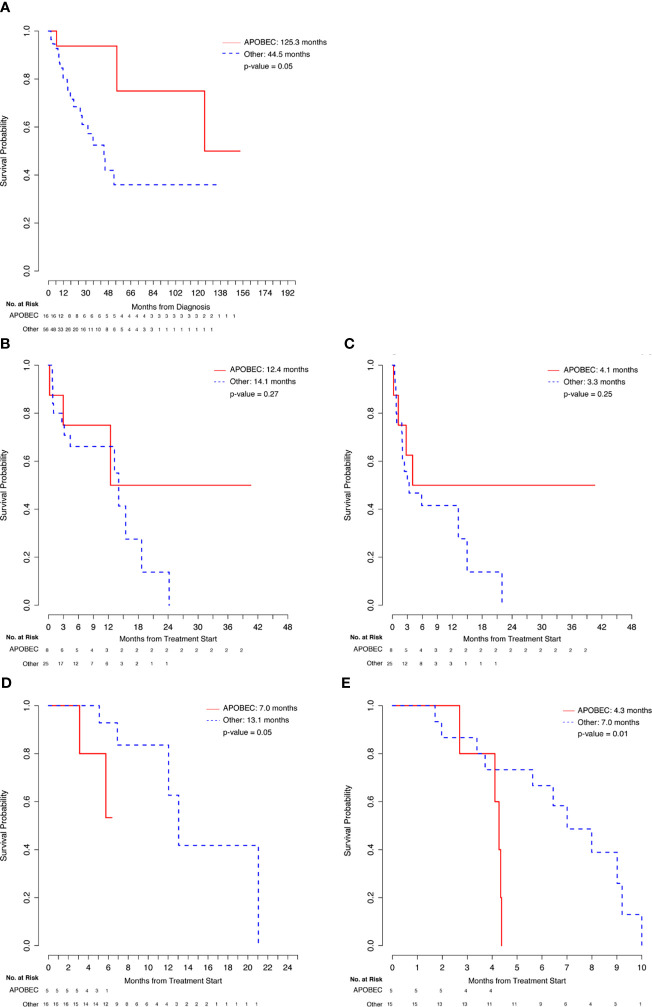
Comparison of APOBEC and Other patients. **(A)** Overall Survival from Diagnosis. **(B)** Overall Survival from Immunotherapy Start. **(C)** Progression-Free Survival from Immunotherapy Start. **(D)** Overall Survival from Chemotherapy Start. **(E)** Progression-Free Survival from Chemotherapy Start.

There were 16 patients with APOBEC mutational signature and 56 patients in the Other category without a known APOBEC mutational signature (including 27 non-HM patients and 29 patients who were not evaluable for TMB). APOBEC patients had a longer median OS from initial diagnosis relative to Other patients (125.3 months *vs* 44.5 months, p = 0.05) (**Figure 3A** and **Table 4**). About 81% of APOBEC patients and 63% of Other patients were alive at the time of analysis. Of the APOBEC patients, 8 received IO and 5 received chemotherapy.

**Table 4:** Comparison of outcomes in APOBEC vs Other patients.

APOBEC vs. Other			
Patient Group (N)	Comparison	Median Survival (months)	p-value
APOBEC (16) vs Other (56)	OS from initial diagnosis	125.3 vs 44.5	**0.05**
	From IO Start		
APOBEC (8) vs	OS	12.4 vs 14.1	0.27
Other (25)	PFS	4.1 vs 3.3	0.25
	From Chemotherapy Start		
APOBEC (5) vs	OS	7.0 vs 13.1	**0.05**
Other (15)	PFS	4.3 vs 7.0	**0.01**

HM, hypermutated; NHM, non-hypermutated; OS, overall survival; PFS, progression-free survival; IO, immunotherapy.

Bold: statistically significant.

Comparing the 8 APOBEC patients with the 25 Other patients that were treated with immunotherapy regimens, although evaluable APOBEC patients had numerically higher ORR to IO treatment (4/7, 57%), relative to evaluable Other patients (8/25, 32%), this difference was not statistically significant. Similarly, the difference in median OS (12.4 *vs*. 14.1, p=0.27) and median PFS (4.1 *vs*. 3.3, p=0.25) were also not statistically significant between these two groups (**Figures 3B, C** and **Table 4**). APOBEC patients received Pembrolizumab (5/8, 63%) and Atezolizumab (3/8, 38%) while Other patients received Pembrolizumab (17/25, 68%), Atezolizumab (4/25, 16%), Nivolumab (2/25, 8%), Nivolumab/Ipilimumab (1/25, 4%), and Durvalumab/Tremelimumab (1/25, 4%).

In comparing chemotherapy treatment outcomes, the ORR were similar among APOBEC (3/5, 60%) and Other (9/15, 60%) patients. Importantly, despite the similarity in response rates, APOBEC patients had shorter median PFS (4.3 *vs*. 7.0, p=0.01) and OS (7.0 *vs* 13.1, p=0.05) relative to the 15 Other patients (**Figures 3D, E** and **Table 4**). APOBEC patients were treated with cisplatin-based chemotherapy (4/5, 80%) and carboplatin-based chemotherapy (1/5, 20%) while Other patients were treated with cisplatin-based chemotherapy (9/15, 60%), carboplatin-based chemotherapy (5/15, 33%), capecitabine (1/15, 7%).

## Discussion

In this large cohort of patients with aUC whose tumors were all assessed with the UCSF500 test, our institutional DNA-based NGS panel, a significant proportion of evaluable tumors were found to have TMB ≥10 mutations/Mb and all of the hypermutated tumors evaluable for mutational signatures were found to have APOBEC mutational signatures. Increased tumor mutational burden and presence of APOBEC mutational signatures were both found to be prognostic of improved overall survival in this patient population. Neither of these biomarkers were predictive of outcomes with immune checkpoint inhibitor treatment. However, both hypermutated status and presence of APOBEC mutational signatures were associated with inferior outcomes with platinum-based chemotherapy treatment, nominating them as potential negative predictive markers for aUC patients treated with chemotherapy.

Urothelial cancers are frequently amongst the most highly mutated tumors (24). In the IMvigor211 clinical trial of patients with platinum-refractory urothelial carcinoma, the median TMB in tumors was 9.6 mutations/Mb (11). This high mutation burden in urothelial carcinoma is frequently driven by the genetic instability caused by APOBEC mutagenesis (23). As an example, in the IMvigor130 clinical trial, presence of APOBEC mutational signatures was associated with higher TMB (25). However, urothelial carcinomas have high mutational complexity and alternate mechanisms of mutagenesis that can account for a high TMB. Thus, it is not necessarily the case that urothelial tumors with a high TMB overlap completely with tumors harboring APOBEC mutational signatures and the two can be used as distinct biomarkers. In this dataset however, among 19 tumors with high TMB, all 16 tumors evaluable for mutational analysis were found to have APOBEC mutational signatures. Although the overall numbers were too small to draw definitive conclusions, the most common mutations present across the different subsets of HM and non-HM, as well as APOBEC and non-APOBEC tumors were fairly consistent. Most common mutations included *TERTp* and *TP53* in all groups. Importantly, the targetable *FGFR3* alterations were more commonly found in non-HM and non-APOBEC tumors. This is consistent with previously reported findings showing APOBEC-low tumors to have more *FGFR3* mutations, while APOBEC-high tumors are more likely to have mutations in DNA damage response genes and chromatin regulatory genes (17). All this suggests that the population included in this analysis was fairly representative of the patterns of mutational complexity found in bladder cancer.

Our initial hypothesis was that presence of TMB and APOBEC signatures would be independently associated with more favorable clinical outcomes. Prior studies have suggested that higher TMB levels are associated with improved overall survival and decreased recurrence rates in patients with muscle-invasive bladder cancer (8). Presence of APOBEC signatures was also found to be associated with improved overall survival in muscle-invasive bladder cancer (17). Our findings in this study based on samples obtained from patients with metastatic disease as well as localized disease were overall in agreement with this prior data, as we found improved OS for high TMB patients relative to low TMB patients and for APOBEC patients versus patients without APOBEC mutational signatures. The potential genomic instability reflected by high TMB and associated with APOBEC signatures may account for these improved outcomes leading to the tumor being more vulnerable to immune surveillance. Median overall survival from initial diagnosis and independent of treatment was longer in all comparison groups of this dataset than a median OS that would be expected from a purely metastatic urothelial cancer cohort timed from initial metastatic disease diagnosis. This was due to the inclusion of patients with MIBC who had curative intent treatment and never had progression to metastatic disease as well as inclusion of patients who were initially diagnosed with localized disease many years prior to eventual progression to metastatic disease.

We additionally hypothesized that increased TMB and presence of APOBEC mutational signatures would be predictive of responses to immune checkpoint inhibitors. Analyses from clinical trials in urothelial carcinoma have indicated that APOBEC mutational signatures and high TMB are both associated with improved outcomes with immunotherapy agents including both anti-PD-1 antibody nivolumab and anti-PD-L1 antibody atezolizumab (9, 11, 16, 25). The genomic instability and plethora of mutations in the tumor associated with these biomarkers are thought to be the mechanisms underlying enhanced response to immune checkpoint inhibitors. While we did not see this association in the current analysis, this may have been the consequence of relatively small numbers being compared. Other biomarkers, such as PD-L1 status, may have served as additional confounders leading to these findings not being observed in the current dataset. Most patients included in our analysis had unknown PD-L1 status. However, for patients with available PD-L1 status, there was no significant difference in PD-L1 expression across the subgroups being compared.

The potential impact of TMB and APOBEC as biomarkers of response to chemotherapy in bladder cancer was less clear from the outset based on previously available data. Prior studies have suggested that APOBEC-induced mutagenesis is clonally enriched in chemotherapy-treated urothelial cancer (26). A study in a cohort of 73 aUC patients subsequently treated with chemotherapy showed increased expression of APOBEC mRNA in tumor samples to be associated with longer OS (27). However no data regarding APOBEC mutational signatures as predictors of response to chemotherapy were previously available. TMB status was likewise not shown to be predictive of responses to second-line chemotherapy in the Keynote-045 trial with Pembrolizumab in platinum-refractory urothelial cancer (13). Due to the limitations of these data we hypothesized that neither high TMB, nor presence of APOBEC mutational signatures would be predictive of responses to chemotherapy. However, the analysis presented here revealed that high TMB and presence of APOBEC mutational signatures were both associated with inferior chemotherapy outcomes. These inferior outcomes were not readily explained by other potential prognostic or predictive genomic biomarkers in this patient population. Among all patients who received chemotherapy there were no appreciable differences, in either the HM *vs* NHM or Apobec *vs* Other analyses, between the comparison subgroups in terms of tumor alterations in TP53, RB1 or DNA damage repair (DDR) genes. Consequently, our analysis is the first to nominate high TMB and presence of APOBEC mutational signatures as negative predictive biomarkers of chemotherapy response in patients with aUC. Since these tumors are more highly mutated, they are likely more aggressive and, as a result, may not respond to traditional chemotherapy treatment. While the mechanism explaining these findings is not immediately clear, a potential explanation may be that chemotherapy treatments are causing immune cell depletion in these otherwise immunologically responsive tumors. Alternatively, a clonal selection of more aggressive variants in these genomically unstable tumors may be taking place under selective pressure from chemotherapy treatment. These potential mechanisms should also be further assessed in future studies.

While retrospective data presented in this manuscript should be further validated, conclusions derived from these findings can help inform clinical decision making and clinical trial design for patients with aUC. Based on this data, it can be surmised that patients with high TMB and APOBEC mutational signatures may have better outcomes overall but may also not do as well with chemotherapy treatment. Although we did not observe these biomarkers to be predictive of responses to immunotherapy treatments, other studies have suggested improved responses to treatments with both anti-PD-1 and anti-PD-L1 immune checkpoint inhibitors in high TMB patients and patients with APOBEC mutational signatures. Consequently, in the appropriate clinical context, treatments with immune checkpoint inhibitors may be considered in lieu of chemotherapy in selected patients with high TMB or presence of APOBEC mutational signatures.

There were limitations to this study, including the retrospective nature of this analysis, a design which introduces numerous confounders. As an example, the presence of other putative biomarkers, such as PD-L1 status and other unknown genomic alterations, may have confounded the results in this relatively small sample size. Radiographic responses were not reviewed according to strict RECIST criteria but were assessed by a single investigator to help address potential inter-observer heterogeneity. The study was also limited to a single academic center and the findings may be difficult to generalize to other academic institutions or community sites. It is also unknown whether data obtained from UCSF500 platform can be generalized to other NGS platforms. In spite of these limitations, this study presents important hypothesis-generating data that can inform future studies and potentially impact clinical decision making.

## Conclusions

This single-institution retrospective analysis in a large cohort of patients with advanced urothelial cancer, revealed findings that supported prior data nominating high TMB and APOBEC mutational signatures as positive prognostic markers in this patient population. This was additionally the first study to nominate TMB-high status and APOBEC mutational signatures as predictive biomarkers indicating inferior responses to chemotherapy treatment in patients with advanced urothelial cancer. While these findings should be further validated, they can nevertheless inform treatment decisions and clinical trial design for patients with advanced urothelial carcinoma in the appropriate clinical context.

## Data Availability Statement

The original contributions presented in the study are included in the article/supplementary material. Further inquiries can be directed to the corresponding author.

## Ethics Statement

The studies involving human participants were reviewed and approved by University of California, San Francisco Institutional Review Board. Written informed consent for participation was not required for this study in accordance with the national legislation and the institutional requirements.

## Author Contributions

Concept: DN and VK. Data collection: DN, LZ, HM, TJ, PD, BS, JG, JZ, NJ, SU, CO, EC, and VK. Data analysis and interpretation: all authors. All authors contributed to the article and approved the submitted version.

## Conflict of Interest

VK reports serving in a consulting or advisory role for AstraZeneca, Clovis, Janssen, Pfizer, EMD Serono, Seattle Genetics/Astellas, Dendreon, Guidepoint, GLG, and receiving research funding for the institution from Endocyte, Nektar, Clovis, Janssen and Taiho, all unrelated to the current submission. LF reports research support from Abbvie, Bavarian Nordic, Bristol Myer Squibb, Corvus, Dendreon, Janssen, Merck, and Roche/Genentech, and also declares serving as a scientific advisory board member to Actym, Allector, Astra Zeneca, Atreca, Bioalta, Bolt, Bristol Myer Squibb, Immunogenesis, Merck, Merck KGA, Nutcracker, RAPT, Scribe, Senti, Soteria, TeneoBio, and Roche/Genentech, all unrelated to the current submission.

The remaining authors declare that the research was conducted in the absence of any commercial or financial relationships that could be construed as a potential conflict of interest.

The reviewer RG declared a past co-authorship with one of the authors VK to the handling Editor.

## Publisher’s Note

All claims expressed in this article are solely those of the authors and do not necessarily represent those of their affiliated organizations, or those of the publisher, the editors and the reviewers. Any product that may be evaluated in this article, or claim that may be made by its manufacturer, is not guaranteed or endorsed by the publisher.

## Abbreviations

UC, urothelial carcinoma; NMIBC, non-muscle-invasive bladder cancer; MIBC, muscle-invasive bladder cancer; Auc, advanced urothelial cancer; IO, immunotherapy; SOC, standard of care; EV, enfortumab vedotin; NGS, next-generation sequencing; TMB, tumor mutational burden; MSI, microsatellite instability; UCSF, University of California, San Francisco; APOBEC, apolipoprotein B mRNA editing catalytic polypeptide-like; EMR, electronic medical record; IRB, institutional review board; ORR, observed response rate; HM, hypermutated; NHM, non-hypermutated; PFS, progression-free survival; OS, overall survival; DDR, DNA damage repair.
